# Identification of a Five-Gene Signature for Predicting Survival in Malignant Pleural Mesothelioma Patients

**DOI:** 10.3389/fgene.2020.00899

**Published:** 2020-08-07

**Authors:** Yiyang Bai, Xiao Wang, Jia Hou, Luying Geng, Xuan Liang, Zhiping Ruan, Hui Guo, Kejun Nan, Lili Jiang

**Affiliations:** ^1^Department of Medical Oncology, The First Affiliated Hospital of Xi’an Jiaotong University, Xi’an, China; ^2^Oncology Hospital, Xi’an International Medical Center Hospital, Xi’an, China

**Keywords:** malignant pleural mesothelioma, gene expression, signature, overall survival, prognosis

## Abstract

Malignant pleural mesothelioma (MPM), predominantly caused by asbestos exposure, is a highly aggressive cancer with poor prognosis. The staging systems currently used in clinics is inadequate in evaluating the prognosis of MPM. In this study, a five-gene signature was developed and enrolled into a prognostic risk score model by LASSO Cox regression analysis based on two expression profiling datasets (GSE2549 and GSE51024) from Gene Expression Omnibus (GEO). The five-gene signature was further validated using the Cancer Genome Atlas (TCGA) MPM dataset. Univariate and multivariate Cox analyses proved that the five-gene signature was an independent prognostic factor for MPM. The signature remained statistically significant upon stratification by Brigham stage, AJCC stage, gender, tumor size, and lymph node status. Time-dependent receiver operating characteristic (ROC) curve indicated good performance of our model in predicting 1- and 2-years overall survival in MPM patients. The C-index was 0.784 for GSE2549 and 0.753 for the TCGA dataset showing moderate predictive accuracy of our model. Furthermore, Gene Set Enrichment Analysis suggested that the five-gene signature was related to pathways resulting in MPM tumor progression. Together, we have established a five-gene signature significantly associated with prognosis in MPM patients. Hence, the five-genes signature may serve as a potentially useful prognostic tool for MPM patients.

## Introduction

Malignant pleural mesothelioma (MPM), the most common form of malignant mesothelioma, is a highly aggressive neoplasm arising from the pleural mesothelial tissues covering the lung and is predominantly associated with occupational and environmental exposure to asbestos fibers (Wagner et al., 1960; Walker et al., 1983). Centers for Disease Control and Prevention (CDC) reports the annual number of malignant mesothelioma deaths increased by 4.8%, from 2479 in 1999 to 2579 in 2015, in the United States (Mazurek et al., 2017). Though it is still considered a rare disease, as many as 3000 new cases are diagnosed annually in United States alone (Vogelzang et al., 2003). Considering the latency period between the first asbestos exposure to MPM development often ranges anywhere between 20 and 71 years, the global incidence of MPM will likely be on the rise (Lin et al., 2019). Although the time from exposure to onset is long, the progression from onset is rapid. MPM patients often have non-specific symptoms at first which makes diagnosis extremely challenging at early stage. Moreover, the lack of an accurate and universally accepted staging system makes it even harder for investigation and treatment of the disease, leading to the poor prognosis of MPM (Rusch, 1995; Kindler et al., 2018).

Researchers have found several clinicopathological factors associated with poor prognosis of MPM, such as the male gender, elevated serum lactate dehydrogenase levels, chest pain, thrombocytosis, non-epithelial histology, and age > 75 years (Curran et al., 1998; Herndon et al., 1998). Simultaneously, potent biomarkers have been studied in relation to pathogenesis, diagnosis and prognosis of MPM. To date, non-tissue-based biomarkers have been characterized, including soluble mesothelin-related protein (SMRP), osteopontin and fibulin-3 (Hollevoet et al., 2012; Kindler et al., 2018). However, none of these biomarkers being evaluated at this time for MPM have demonstrated sufficiently rigorous prospective or blinded validation to recommend their use (Kindler et al., 2018). Some studies have evaluated the diagnostic value of microRNA to differentiate MPM from normal pleural mesothelial proliferation or other carcinomas (Busacca et al., 2010; Benjamin et al., 2010; Balatti et al., 2011). The six-microRNA signature constructed by Kirschner et al. (2015) was reported to predict the survival of MPM patients. However only a small number of long and short-term survivors were compared following extrapleural pneumonectomy, and normal samples were not included as a control, thereby limiting the use of this specific signature (Kirschner et al., 2015). Furthermore, an increasing number of studies using gene expression profiling have primarily discovered new oncogenes or tumor suppressor genes or have simply used MPM tumor samples without normal samples to construct the prognostic model. For instance, Gordon et al. developed and validated a nine-ratio (six gene) signature to differentially diagnose MPM from adenocarcinoma, and they also defined a molecular classification of MPM using transcriptional profiling by microarray, however did not independently validate the results (Gordon et al., 2002, 2005). The study took inspiration from previous research and concerning the difficulty to apply the MPM staging system into clinical work to make accurate evaluation of prognosis for MPM patients (Rusch et al., 2012), it is of great value to use bioinformatics methods to discover prognostic genes between MPM tumor and normal tissues as possible biomarkers and construct a risk-score model to clarify MPM patients into high- and low-risk subgroups, leveraging an objective approach in MPM patients’ prognosis evaluation.

In this study, based on the gene expression profiling data from GEO and TCGA dataset, we developed and validated a reliable five-gene signature model independent of clinicopathological factors that improved the risk stratification for MPM patients.

## Materials and Methods

### Datasets

The two gene expression arrays of human MPM datasets GSE2549 (Gavin et al., 2005) and GSE51024 (Suraokar et al., 2014a) were derived from the Gene Expression Omnibus (GEO)^1^, which is a database repository of high throughput gene expression data and hybridization arrays, chips, microarrays. GSE2549 and GSE51024 sets were conducted on GPL96 (Affymetrix Human Genome U133A Array) and GPL570 (Affymetrix Human Genome U133 Plus 2.0 Array) platforms respectively. The GSE2549 dataset includes 40 discarded human MPM tumor specimens and 9 normal specimens in which four are normal lung specimens and five are normal pleura. The GSE51024 dataset includes 41 MPM tumor tissues along with 41 paired normal tissues. For the validation of TCGA set, we downloaded the mRNA expression data (RNA-seq in FPKM value) which includes 85 MPM patients together with the clinical information from the Cancer Genome Atlas^2^.

### Differential mRNA Analysis

To identify the differentially expressed genes (DEGs), GSE2549 and GSE51024 MPM datasets were employed. After annotating the probes into gene symbols by the platform annotation files, we used R package LIMMA (Ritchie et al., 2015) to get the DEGs between the tumor and normal tissue following the criteria of adjusted *P*-value < 0.05, |log_2_FoldChange| > 1 in both sets. Finally, by overlapping the upregulated and downregulated DEGs from the two datasets, we got the DEGs in both datasets.

### Construction of the Prognostic Model by LASSO Cox Regression

By performing the univariate Cox regression analysis on the candidate DEGs from the two discovery sets, we calculated the correlation between each gene and the overall survival time of each patient and investigated the genes having strongest association with the patients’ overall survival time following the criteria of *P* < 0.05. R package “survival” was used to perform the univariate Cox regression analysis (Gordon et al., 2020; Terry and Therneau, 2020). The least absolute shrinkage and selection operator (LASSO) Cox regression with 10-time cross validation was used to choose the penalty regularization parameter λ (Gui and Li, 2005). The coefficient of each gene was forced to shrink to zero which eliminated the correlation between the selected genes and prevented the model from being overfitting. By applying the minimum deviance, lamda.min, genes were selected. R package “glmnet” was used to perform LASSO Cox regression analysis (Goeman, 2010; Friedman et al., 2020). Together with the coefficient of each gene generated by multivariate Cox regression analysis, the prognostic risk score model was constructed. R package “survminer” was used to perform the multivariate Cox regression analysis (Kassambara et al., 2020). Based on the expression of each gene discovered, each patient’s risk score was calculated according to the risk score model. The risk score model was then used to evaluate the prognosis of MPM patients.

$$\mathrm{Risk}\,\mathrm{score}=\sum_{i=1}^{n}\mathrm{Coeffcient}\,i^{*}\,\mathrm{Expression}\,\mathrm{of}\,\mathrm{Gene}\,i$$

### Construction of the Prognostic Model by Three Other Methods

To validate the prognostic model constructed by LASSO Cox regression method. Forward stepwise regression, bidirectional stepwise regression and relaxed LASSO methods were introduced to rebuild the model. Prognosis related genes detected by univariate Cox regression analysis were used to perform the analyses for the three methods. In forward stepwise regression analysis, genes were ranked by their z-score, which represented their predictive power. The gene with the highest z-score was first enrolled and gene was subsequently added according to the z-score rank list from high to low. The AIC (Akaike information criterion) for each model was calculated new gene was terminated being added to the model if the AIC stopped decreasing which indicated the genes already enrolled giving the best prognostic performance. The bidirectional stepwise regression procedure included the iterations between the “forward” and “backward” steps. R package “My.stepwise.coxph” was used to do the analysis (My.stepwise.coxph, 2017). We found the R package “fastcox” was for Lasso and Elastic-Net penalized Cox’s regression in high dimensions models and it could help realize the relaxed lasso analysis (Yang and Zou, 2013a, b). The penalty regularization parameter λ was chosen via 10-time cross-validation. By applying the minimum deviance, lamda.min, genes were selected. The genes selected by each of the three methods were then enrolled in the multivariate Cox regression analysis respectively and the risk score models were then established with the method mentioned above.

### Survival Analysis and ROC Analysis

According to the risk score formula, we calculated the risk score for each patient in GSE2549 discovery set and TCGA validation set. Patients from the two datasets were then divided into low-risk and high-risk groups respectively with the median cutoff of the risk score. Kaplan-Meier survival curves were performed to evaluate whether there was significant difference between the low-risk and high-risk groups by log-rank test with *P* < 0.05. Univariate and multivariate analyses were conducted to see whether the five-gene signature could be a prognostic factor for MPM independent of other clinicopathological factors. *P* < 0.05 was considered significant. Hazard ratios and 95% confidence interval were also calculated. Univariate and multivariate analyses were performed using IBM SPSS version 23.0 (IBM Corp., NY, United States). Time-dependent receiver operating characteristic (ROC) curve analyses were conducted to evaluate the prognostic effectiveness of the risk score model compared with other clinicopathological factors and one literature model. We used R package “survivalROC” to perform ROC analysis (Heagerty et al., 2000; Patrick, 2013). C-index was calculated by R package “survival” (Terry and Therneau, 2020). R package “compareC” was used to perform the comparison of C-index value (Le Le Kang, 2015). The method was for statistical comparison of two diagnostic or predictive systems, of which they could either be two biomarkers or two fixed algorithms, in terms of their *C* indices (Kang et al., 2015). Z score test was used for hypothesis testing.

### Functional Enrichment Analysis

Gene Set Enrichment Analysis was conducted between the low-risk and high-risk groups to predict the possible molecular mechanisms responsible for the poor prognosis of MPM. Molecular Signatures Database (MSigDB) C2 Canonical pathways gene set database was used to screen the significant pathways with the criteria of |NES| (normalized enrichment score) > 1, NOM *P* < 0.05 and FDR (false discovery rate) *q* < 0.05 after performing 1000 permutations (Liberzon et al., 2011). The enrichment analysis was performed by GSEA 4.0.3^3^.

## Results

### Identification of Candidate DEGs

**FIGURE 1:**
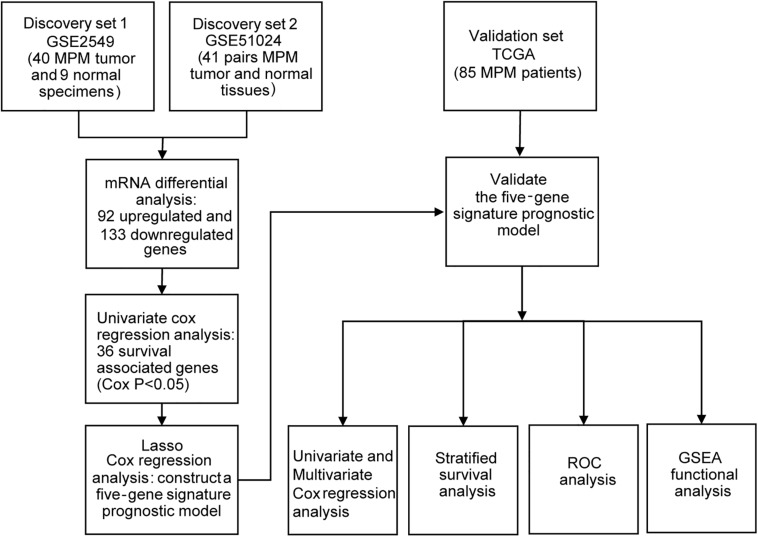
Flowchart of the study design.

**FIGURE 2:**
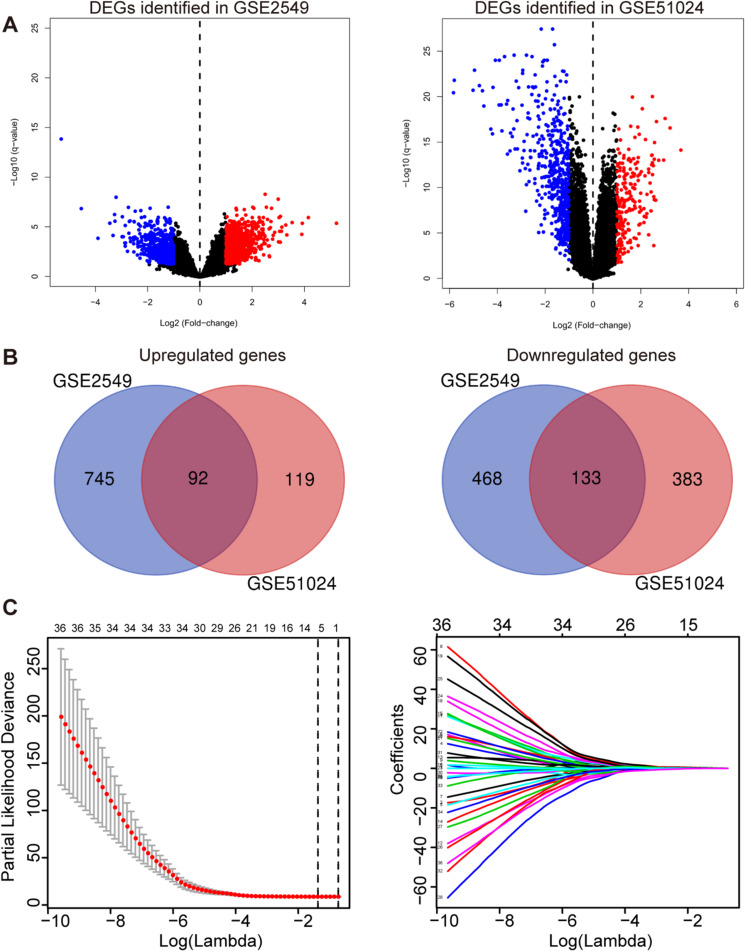
Construction of the five-gene risk score model. **(A)** The volcano plots of GSE2549 and GSE51024 showing the DEGs between MPM tumor and normal tissues. |log_2_ Fold Change| > 1, *q*- < 0.05 for DEGs. Blue dot represents the significant downregulated genes and red dot represents the significant upregulated genes. **(B)** Venn of the DEGs in gene expression datasets GSE2549 and GSE51024. **(C)** Left panel: Plots of the cross-validation error rates. The two vertical dotted line represent the largest lambda value with minimum error (left) and 1- standard error (right); Right panel: LASSO coefficient profiles of the 36 genes detected by univariate cox regression analysis.

To obtain the differentially expressed genes (DEGs) between human MPM tumor and normal tissues, two expression datasets GSE2549 and GSE51024 were enrolled as discovery datasets (Figure 1). We firstly screened for the DEGs between MPM tumor and normal tissues in these two datasets using LIMMA analysis in R (*q* < 0.05, |log_2_ Fold Change| > 1). A total of 1438 DEGs, including 837 upregulated genes and 601 downregulated genes, were identified in GSE2549 between 40 MPM tumor specimens and 9 normal specimens. A total of 727 DEGs, including 211 upregulated genes and 516 downregulated genes were screened in GSE51024 between 41 MPM tumor tissues and 41 paired normal tissues. The volcano plots were generated for both datasets (Figure 2A). Moreover, 92 upregulated genes and 133 downregulated genes overlapped between GSE2549 and GSE51024 (Figure 2B). Therefore, a total of 225 candidate DEGs were selected.

### Construction and Validation of the Prognostic Risk Score Model for MPM

By performing univariate Cox regression analysis between these 225 candidate DEGs and survival data of discovery set GSE2549, 36 genes were detected (*P* < 0.05). A LASSO Cox regression analysis together with 10-time cross validation was then conducted to eliminate the number of genes and select those with non-zero coefficient (Figure 2C). Five genes were identified and using multivariate Cox regression analysis, the coefficient of each gene was calculated. Therefore, a five-gene signature risk score model was developed based on the five genes along with their coefficients and gene expression level. Risk score = 0.1197 × expression of CDH2 + 0.6824 × expression of CKS2 + 0.5594 × expression of KIF11 + 0.7141 × expression of KIF18B + 0.5004 × expression of LOX. To confirm the validity of the signature, we used three other methods, forward stepwise regression, bidirectional stepwise regression and relaxed LASSO, to construct the prognostic models and compare them with the five-gene signature. We found the genes selected by the three methods overlapped with the five-gene signature which confirmed the validity of the genes selected by LASSO Cox regression method (Supplementary Tables 1, 2 and Supplementary Figure 1). We then used AIC, C-index and ROC curves to evaluate and compare between the models and found the five-gene model showed overall better performance (Supplementary Tables 2, 3 and Supplementary Figure 2). We further validated the differential expressions of the five genes between tumor and normal tissues in GSE2549 and GSE51024 sets. All five genes were significantly overexpressed in tumor tissues (Supplementary Figure 3A). At the same time, we did the Kaplan-Meier curve to evaluate the relationship between the gene expression and the overall survival (OS). The five genes were strongly negatively related to the OS in GSE2549 dataset (Supplementary Figure 4).

**FIGURE 3:**
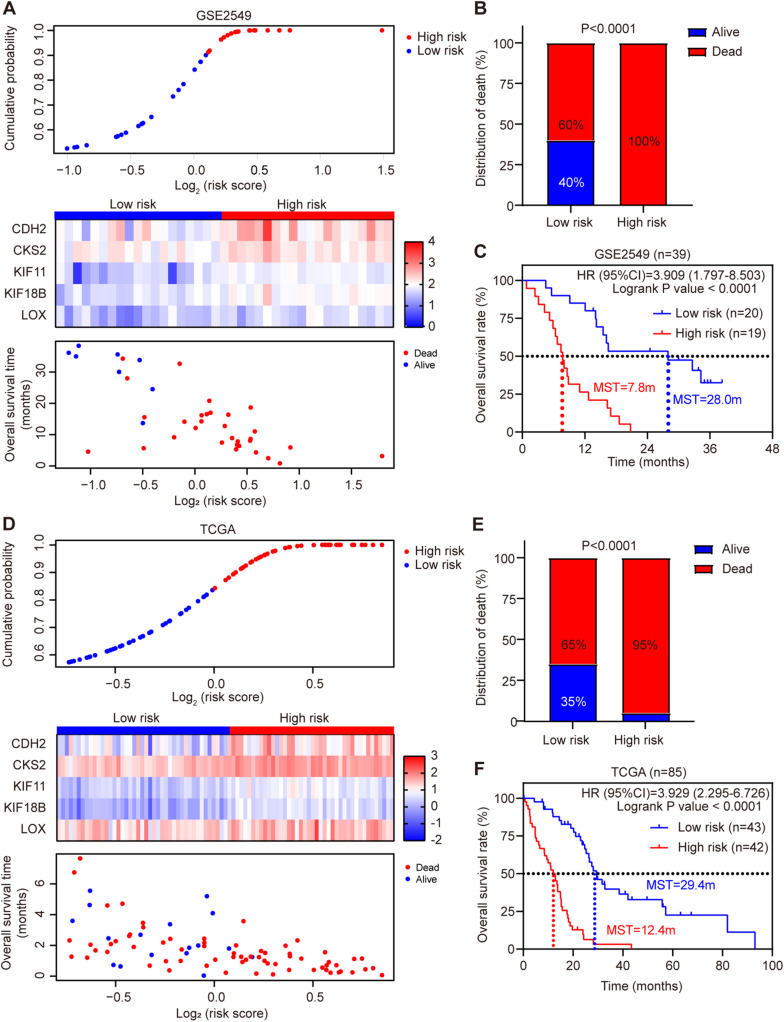
Risk score distribution and validation of the five-gene signature risk score model in GSE2549 and TCGA datasets. **(A,D)** Upper panel: Low-risk and high-risk score distribution. Middle panel: the heatmap showing the five-gene expression profiles for each patient in low-risk and high-risk subgroups; Lower panel: the distribution of risk score with patient survival status. **(B,E)** Comparison of the MPM death rate in low-risk and high-risk groups. The death rate was higher in high-risk group than low-risk group. *P*-value was calculated by Chi-square test. **(C,F)** Kaplan-Meier survival analysis for the low-risk and high-risk patients (log-rank test).

Each patient’s risk score was calculated according to the risk score model in the discovery cohort GSE2549. The patients were divided into low-risk or high-risk group by the median risk score. Patients in high-risk group had much shorter survival time than those in low-risk group (hazard ratio = 3.909, 95% CI = 1.797–8.503, log-rank test *P* < 0.0001). Chi-square analysis showed the death rate was significantly higher in high-risk group than low-risk group (Figures 3A–C). Simultaneously, we used MPM TCGA dataset (85 cases) as a validation cohort to confirm the reproducibility of the risk score model (Figures 3D–F). The prognostic signature was successfully validated in TCGA dataset showing that patients in high-risk group had significantly shorter OS than low-risk group patients (hazard ratio = 3.929, 95% CI = 2.295–6.726, log-rank test *P* < 0.0001). In addition, all five genes were validated significantly negatively related to the OS in TCGA dataset (Supplementary Figure 5).

To evaluate whether the five-gene signature could be an independent prognostic factor for MPM, we conducted the univariate and multivariate Cox regression analyses in both GSE2549 and TCGA datasets. From the univariate analysis of GSE2549 dataset in Table 1, we observed that patients with mixed histological subtype, lymph node positive or high risk score were more likely to have shorter survival time and worse prognosis compared to patients with epithelial subtype, lymph node negative or low-risk score. Hence, these results showed that histological subtype, node status and risk score had a relatively significant impact on prognosis of MPM patients. From the univariate analysis of TCGA dataset in Table 2, risk score was the only factor found to influence the prognosis of MPM patients. After controlling for gender, race, histological type, Brigham stage, AJCC stage, tumor size, lymphatic metastasis status and organ metastasis status, the multivariate analysis results showed the five-gene signature remained an independent prognostic factor in both datasets (*P* < 0.001 in Table 1, *P* < 0.0001 in Table 2).

**TABLE 1:** Univariate and multivariate survival analysis of GSE2549.

	Univariate analysis			Multivariate analysis	
Variable	N	*P*-value	HR (95% CI)	*P*-value	HR (95% CI)
**Histological subtype**					
Epithelial	23	**0.038**	2.232 (1.047–4.759)	0.717	0.834 (0.312–2.226)
Mixed	16				
**Node status**					
N0	8	**0.045**	2.945 (1.025–8.461)	0.089	3.060 (0.843–11.106)
N +	31				
**Margins**					
Negative	5	0.347	1.771 (0.538–5.832)	0.179	2.703 (0.634–11.529)
Positive	34				
**Brigham stage**					
Stage1 + 2	12	0.394	1.404 (0.644–3.064)	0.628	0.784 (0.292–2.101)
Stage3 + 4	27				
**Risk score**					
Low-risk	20	**<0.0001**	4.963 (2.190–11.247)	**<0.001**	5.995 (2.178–16.502)
High-risk	19				

N, number; HR, hazard ratio; CI, confidence interval. *One patient was not included in the univariate and multivariate analysis of the GSE2549 dataset, as the clinical information was not available. All P values in bold were less than 0.05 which were considered statistically significant.

**TABLE 2:** Univariate and multivariate survival analysis of TCGA.

	Univariate analysis			Multivariate analysis	
Variable	N	*P*-value	HR (95% *CI*)	*P*-value	HR (95% *CI*)
**Gender**					
Female	11	0.302	0.690 (0.340–1.397)	**0.011**	0.354 (0.159–0.785)
Male	45				
**Race**					
Asian	1	0.518	0.719 (0.264–1.958)	0.924	0.948 (0.317–2.836)
White	55				
**AJCC stage**					
I + II	18	0.621	0.850 (0.447–1.617)	0.311	1.942 (0.538–7.017)
III + IV	38				
**T stage**					
T1 + T2	28	0.455	0.798 (0.441–1.444)	0.179	0.488 (0.171–1.389)
T3 + T4	28				
**N stage**					
N0	33	0.171	0.648 (0.349–1.206)	0.116	0.493 (0.204–1.190)
N +	23				
**M stage**					
M0	53	0.387	1.888 (0.447–7.983)	0.179	3.585 (0.556–23.106)
M1	3				
**Risk score**					
Low-risk	26	**<0.0001**	6.412 (2.806–14.625)	**<0.0001**	9.009 (3.644–22.274)
High-risk	30				

N, number; HR, hazard ratio; CI, confidence interval; AJCC, American Joint Commission on Cancer. *Nineteen patients were not enrolled in the univariate and multivariate analysis of TCGA dataset, as some clinical information was not available. All P values in bold were less than 0.05 which were considered statistically significant.

### Stratified Survival Analysis of the Five-Gene Risk Score Model

**FIGURE 4:**
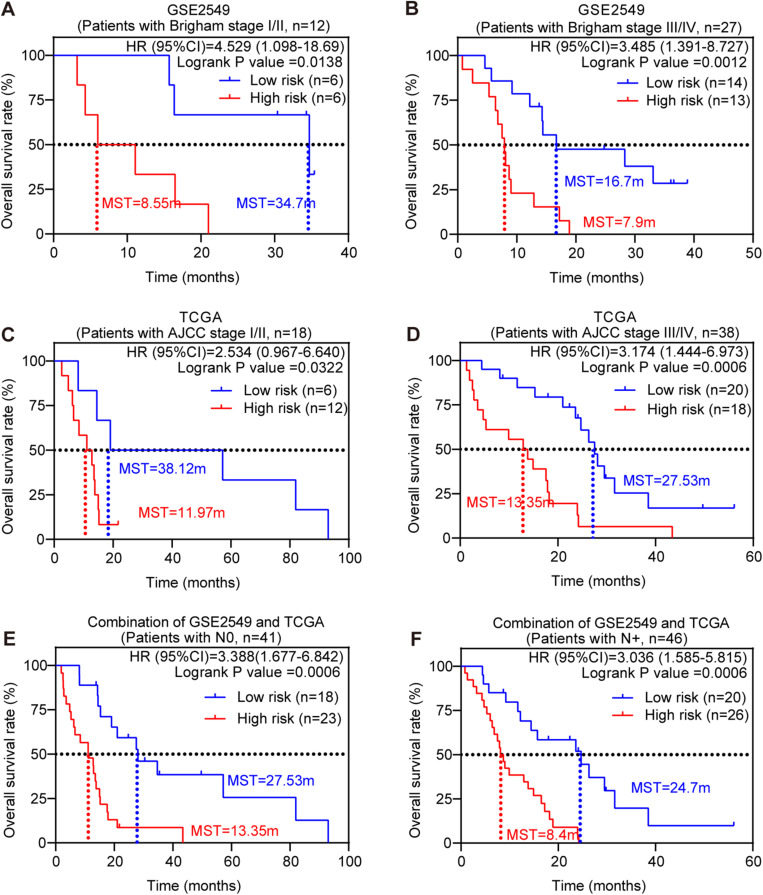
Stratified survival analysis of the five-gene risk score model. Kaplan-Meier survival analysis for the patients with different clinical variables classified by the five-gene risk score model. **(A)** Patients in Brigham stage I/II; **(B)** patients in Brigham stage III/IV; **(C)** patients in AJCC stage I/II. **(D)** Patients in AJCC stage III/IV; **(E)** patients with lymph node negative; **(F)** patients with lymph node positive. *P*-values were calculated by Log-rank test.

Stratified survival analysis was further conducted in subgroups of patients with different clinical variables (gender, histological subtype, tumor size, lymph node metastasis status, AJCC stage, and Brigham stage). According to Tables 3, 4, the five-gene risk score model was generally of statistically significant prognostic value. The MPM patients stratified by Brigham staging system, in either early stage or advanced stage, could be separated into the subgroups of better prognosis and poorer prognosis by the five-gene signature (Figures 4A,B). The model retained its prognostic value when stratified by AJCC staging system in TCGA dataset (Figures 4C,D). At the same time, we also found significant prognostic value of our model in lymph node negative or positive patients in both datasets (Figures 4E,F).

**TABLE 3:** Stratified survival analysis of GSE2549.

Variable	Low risk	High risk	*P*-value	HR (95% CI)
**Histological subtype**				
Epithelial	15	8	**0.0006**	4.444(1.254−15.75)
Mixed	5	11	0.5396	1.390(0.4759−4.058)
**N stage**				
N0	6	2	**0.0473**	5.486(0.3636−82.78)
N +	14	17	**0.001**	3.242(1.465−7.171)
**Brigham stage**				
I + II	6	6	**0.0138**	4.529(1.098−18.69)
III + IV	14	13	**0.0012**	3.485(1.391−8.727)

HR, hazard ratio; CI, confidence interval. *One patient was not included in the stratified analysis of GSE2549 dataset, because the clinical information was not available. All P values in bold were less than 0.05 which were considered statistically significant.

**TABLE 4:** Stratified survival analysis of TCGA.

Variable	Low-risk	High-risk	*P*-value	HR (95% CI)
**Gender**				
Female	5	6	**0.0123**	3.806 (0.961–15.08)
Male	21	24	**0.0001**	3.110 (1.559–6.204)
**AJCC stage**				
I + II	6	12	**0.0322**	2.534 (0.967–6.640)
III + IV	20	18	**0.0006**	3.174 (1.444–6.973)
**T stage**				
T1 + T2	12	16	**0.0018**	2.889 (1.268–6.582)
T3 + T4	14	14	**0.0025**	
**N stage**				
N0	12	21	**0.0017**	2.823 (1.372–5.806)
N +	14	9	**0.0013**	3.937 (1.163–13.33)

HR, hazard ratio; CI, confidence interval; AJCC, American Joint Commission on Cancer. *Nineteen patients were not included in the stratified analysis of TCGA dataset, as some clinical information was not available. All P values in bold were less than 0.05 which were considered statistically significant.

### Comparison of Five-Gene Risk Model With Other Clinicopathological Factors and Literature Models

**FIGURE 5:**
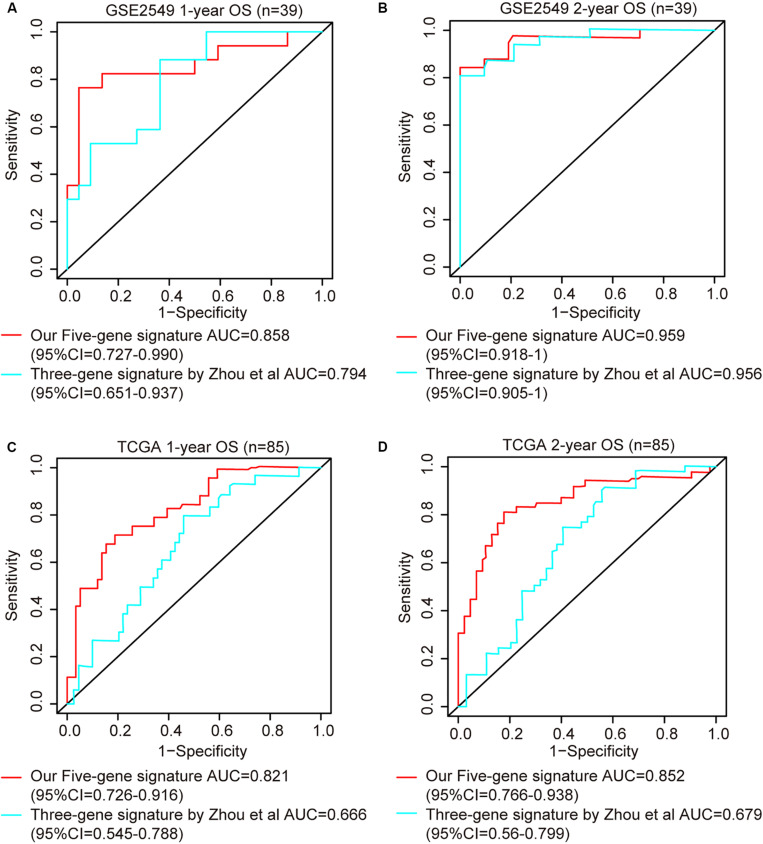
ROC curves of five-gene risk score model compared with the literature model by Zhou et al. (2019). The *X* axis indicates false positive rate. The *Y* axis indicates true positive rate. One patient in GSE2549 dataset was not enrolled in the analysis because the clinical information was not available. **(A)** 1-year OS in GSE2549; **(B)** 2-year OS in GSE2549; **(C)** 1-year OS in TCGA; **(D)** 2-year OS in TCGA.

ROC analysis was conducted to evaluate the efficiency of our five-gene risk score model. The AUCs (area under curve) for the 1- and 2-years OS in GSE2549 dataset were 0.858 (95% CI = 0.727–0.990) and 0.959 (95% CI = 0.918–1) (Figures 5A,B). We verified the efficiency using TCGA dataset. The AUCs for 1- and 2-years OS were 0.821 (95% CI = 0.726–0.916) and 0.852 (95% CI = 0.766–0.938) (Figures 5C,D). Except for the 5-gene signature, we also did the ROC analyses of the clinicopathological factors available in either dataset (Supplementary Figures 6, 7). To further evaluate the prognostic value of the five-gene signature model, we compared our model with a three-gene prognostic model established by Zhou et al. (2019). The AUCs for 1- and 2-years OS in either GSE2549 or TCGA dataset by our model were comparable with Zhou’s model. We also used concordance index (C-index) to evaluate our model. And R package “compareC” was used to do the statistical comparison of the C-indices between our model and Zhou’s model. The C-indices for GSE2549 dataset were comparable between our model and Zhou’s model and no statistical difference was detected (Table 5). However, our model showed significant higher C-index value than Zhou’s model (*P* < 0.05) in TCGA validation dataset (Table 6).

**TABLE 5:** C-index comparison in GSE2549 dataset.

Signature	C-index	Z-score	*P*-value
Our five-gene signature	0.784 (95% CI = 0.674–0.894)	−0.35839	0.720051
Three-gene signature by Zhou et al. (2019)	0.770 (95% CI = 0.725–0.815)		

**C-index, concordance index; CI, confidence interval.**

**TABLE 6:** C-index comparison in TCGA dataset.

Signature	C-index	Z-score	*P*-value
Our five-gene signature	0.753 (95% CI = 0.698–0.808)	−3.41016	**0.000649**
Three-gene signature by Zhou et al. (2019)	0.608 (95% CI = 0.567–0.649)		

**C-index, concordance index; CI, confidence interval.* All P values in bold were less than 0.05 which were considered statistically significant.*

### Functional Analysis

To obtain the potential biological function of the five-gene signature in MPM tumorigenesis, the Gene Set Enrichment Analysis (GSEA) was conducted to identify the associated pathways between the high-risk and low-risk subgroups in GSE2549 discovery dataset and TCGA validation dataset. Here we used the canonical pathway gene set from the Molecular Signatures database as our gene set database. The gene sets were considered significantly enriched when the definite value of normalized enrichment score was more than 1, Nominal *P*-value was less than 0.005 and FDR *q*-value was less than 0.05. From the GSEA report, we discovered that “RHO GTPASES activate formins,” “Mitotic spindle checkpoint,” “PLK1 pathway,” and “resolution of sister chromatid cohesion” pathways were significantly enriched in high-risk group patients from both discovery and validation sets. Simultaneously, several cancer related pathways such as “TP53 regulates transcription of cell cycle genes,” “regulation of TP53 activity through phosphorylation,” “cell cycle checkpoints,” “kinesins,” and “DNA double strand break repair” were also enriched in the high-risk group of TCGA. They were all reported to be involved in tumorigenesis and tumor progression (Supplementary Figure 8).

## Discussion

Over the years, several staging systems have been proposed for MPM (Rusch and Venkatraman, 1996). The TNM staging system proposed by the International Mesothelioma Interest Group (IMIG) subsequently accepted by the American Joint Commission on Cancer (AJCC) and the Union for International Cancer Control (UICC) is the one that has been generally accepted. Nevertheless, the current AJCC/UICC staging classification for MPM is still difficult to apply to clinical staging and thus may be imprecise in predicting prognosis and providing appropriate treatment for MPM patients (Rusch et al., 2012). Therefore, it is of great significance to explore biomarkers with optimal prognostic value for MPM patients. Research shows that combination of multiple biomarkers will improve the prognostic value instead of a single biomarker (Nalejska et al., 2014). In this study, we developed and validated a five-gene signature to evaluate the prognosis of MPM.

First, we identified 225 candidate DEGs based on two expression microarrays from GEO database. Aiming to eliminate the correlation between the genes selected by univariate analysis and improve the applicability in clinical practice, a five-gene signature risk score model was constructed by LASSO Cox regression. The LASSO Cox regression model has been widely applied to the Cox proportional hazard regression model for survival analysis with high dimensional data (Tibshirani, 1997; Zhang and Li, 2007; Wei et al., 2015; Lin et al., 2020). By studying Hastie et al’s research work on comparing the LASSO with other model selection methods (Hastie et al., 2017), we introduced three more methods to confirm the validity of the genes selected by LASSO Cox regression. The performances of the models were evaluated by AIC, C-index and AUC with respect to the degree of goodness-to-fit, the prediction accuracy and the predictive capacity. From the results, we could see the bidirectional stepwise regression showed the worst performance among these four methods (Supplementary Tables 2, 3 and Supplementary Figure 2). Our five-gene model by LASSO showed better or equivalent performance when compared with the models constructed by forward stepwise regression or relaxed LASSO. At the same time, we noticed four of the five genes (KIF18B, CKS2, LOX, and CDH2) in forward stepwise regression model and four of seven genes (CKS2, KIF11, KIF18B, and LOX) in relaxed LASSO model were overlapped with the five genes in LASSO model which further confirmed the validity of the genes selected by LASSO Cox regression method. Compared with the model constructed by relaxed LASSO method which included seven genes, our signature only had five genes and it showed better or equivalent prediction performance. The general principle for model selection was that for a given level of accuracy, a simpler or a more parsimonious model is preferable to a more complex one (Bozdogan, 1961; Stone, 1981). Taking into account the measurement cost to implement the model and the complexity of the model, the five- gene signature not only had better or equivalent prediction performance but also had higher practicability value.

The model was validated in GSE2549 and TCGA datasets showing that high-risk group patients always had worse clinical outcome and shorter survival time than low-risk group patients. In stratified analysis, our model had good performance in predicting OS. The univariate and multivariate analyses indicate our five-gene signature to be an independent prognostic factor for MPM patients. Simultaneously, our results showed that histological subtype, gender, and lymph node had relatively significant impact on prognosis, and these results were in agreement with many other researches for prognostic factors (Spirtas et al., 1988; Anand, 1994; Curran et al., 1998; Vigneswaran et al., 2017). To evaluate the accuracy and discrimination power of our risk score model, we did the ROC and C-index analyses. Simultaneously, we compared our model with available clinicopathological factors. Our risk score model exhibited stable predictive performance compared with clinicopathological factors (Supplementary Figures 6, 7). Considering our risk score model was using relatively objective gene expression levels tested by microarray or RNAseq while evaluating the prognosis of MPM patients, some of the clinicopathological factors might be variable in evaluation of prognosis of MPM patients. And this might also be due to lack of the data. We also compared our model with the literature model by Zhou et al. (2019), the AUCs for 1- and 2-years OS were comparable in discovery GSE2549 dataset. While putting the two models in the larger validation TCGA dataset, although our model showed higher AUCs, but the confidence of intervals overlapped, indicating no statistical difference. Therefore, we could not determine yet which model was better at evaluating the prognosis of MPM patients from the statistical perspective. Maybe due to the lack of data, we could not detect the statistical difference of AUCs between our model and Zhou’s model. When we used C-index to compare our model and Zhou’s model in the validation TCGA dataset, our model showed higher C-index value with a *P*-value of 0.000649.

To gain more insights into the modulatory roles of the five genes in the signature, GSEA analysis was performed and showed that the “RHO GTPASES activate formins,” “mitotic spindle checkpoint,” “PLK1 pathway,” and “resolution of sister chromatid cohesion” pathways were significantly associated with poor prognosis in high-risk subgroup MPM patients. The Rho family of GTPases is a family of small signaling G protein that regulate actin cytoskeleton organization and dynamics (Vega and Ridley, 2008). Nearly 75% of MPM cases harbor loss of function of core components of the Hippo pathway, which negatively regulates YAP activity (Zhang et al., 2010; Murakami et al., 2011). RhoA may strongly enhance YAP/TAZ activity, thereby promoting the proliferation of MPM in the sense that high YAP/TAZ activity is positively related to high proliferation capacity for MPM (Dupont et al., 2011; Mizuno et al., 2012). Zhang et al. reported a proliferation inhibitory effect in MPM cell lines with GSK269962A, a selective inhibitor of Rho−Kinase (Zhang et al., 2017). These studies, together with our result, indicated that the “Rho GTPASES activate formins” pathway is likely a significant mechanism in MPM tumorigenesis, and might serve as a target in the treatment of MPM patients. Additionally, the “mitotic spindle checkpoint” and “resolution of sister chromatid cohesion” pathways are closely related. The spindle checkpoint delays sister chromatid separation until all chromosomes have undergone bipolar spindle attachment. Dysfunction of this checkpoint contributes to tumorigenesis. Moreover, certain components of the mitotic spindle checkpoint pathway, including CHEK1, BUB1, and MAD2L1 were found to be upregulated in MPM tumors via microarray technology (Crispi et al., 2009). Similarly, Suraokar et al. carried out a gene expression microarray experiment on 53 surgically resected MPMs tumors along with paired normal tissues and found that the mitotic spindle checkpoint pathway was the most significantly altered pathway in MPM patients (Suraokar et al., 2014b). They also evaluated the indicator for the deregulated expression of the mitotic spindle checkpoint pathway in an independent cohort of 80 MPM tumors and found higher nuclear MAD2L1 expression associated significantly (*P* = 0.043) with lower rates of OS. These findings, along with our result, clearly demonstrate the important role of the “mitotic spindle checkpoint” pathway in MPM that the pathway, suggesting that it might be a possible target for MPM therapy. Lastly, PLK1 (polo-like kinase 1), has been identified as a candidate therapeutic target and independent prognostic marker of MPM by an RNAi-based screening study (Linton et al., 2014). The inhibitory effect on cell proliferation following treatment with the PLK1 small inhibitors BI6727, and BI2539 or the PLK1-specific siRNA and artificial microRNA, have been validated in multiple MPM cell lines (Linton et al., 2014; Kato et al., 2016b). Hence, under our five-gene risk score classification, the high-risk MPM patients may benefit from PLK1 specific small molecule inhibitors, which are currently considered to be attractive therapeutic strategies against specific tumor types such as leukemia and non-small cell lung cancer (Medema et al., 2011; Lee et al., 2015).

All five genes (*CDH2*, *CKS2*, *KIF11*, *KIF18B*, and *SEMA3G*) in our model were confirmed to be significantly associated with the OS of MPM patients. Cadherin2 (*CDH2*), encoding N-cadherin protein, is closely related to the epithelial–mesenchymal transition (EMT) process and demonstrated prognostic significance in MPM (Schramm et al., 2010). It was reported that there was a substantial switch from epithelial markers such as E-cadherin and β-catenin to mesenchymal markers such as N-cadherin through epithelium to biphasic and sarcomatoid subtypes, indicating the three histological subtypes of MPM are the consequences of different steps in an EMT process, of which the sarcomatoid subtype has the worst OS (Fassina et al., 2012). Consistent with these previous studies, we found the expression of CDH2 was higher in MPM tumor tissues than normal tissues and the high expression level of CDH2 was associated with poor prognosis (Supplementary Figures 3, 4A, 5A).

Cyclin-dependent kinases regulatory subunit 2 (*CKS2*) is the member of cell cycle dependent protein kinase subunits family. Although rarely reported with MPM, there is accumulating evidence showing CKS2 is upregulated in many types of tumor as a prognostic factor, including hepatocellular carcinoma, colorectal cancer, bladder cancer, breast cancer, gastric cancer and epithelium ovarian cancer (Kawakami et al., 2006; Shen et al., 2010; Tanaka et al., 2011; Yu et al., 2015; Huang et al., 2019; Xu et al., 2019). Our results revealed that the expression of CKS2 was elevated in MPM tumor tissues compared to normal tissues and it was harmful for the prognosis of MPM patients, which is in line with these previous findings of other types of tumor (Supplementary Figures 1, 4B, 5B). Investigators also found that CKS2 may advance tumor progression by promoting tumor cell proliferation and regulating apoptosis (Shen et al., 2013).

Kinesin family member 11 (*KIF11*, also known as *EG5*) and Kinesin family member 18B (*KIF18B*) are two kinesin superfamily members, yet they are from two different kinesin subfamilies and function differently (Lawrence et al., 2004). KIF11 is essential for cell growth and proliferation, involved in the formation of the bipolar spindle in cell mitosis. KIF11 was found overexpressed not only in MPM human tumor samples and MPM human cell lines, but also in blast crisis chronic myelogenous leukemia and pancreatic cancer (Nowicki et al., 2003; Liu et al., 2010; Kato et al., 2016a). This is consistent with our analysis that KIF11was significantly overexpressed in MPM tumor tissues and was confirmed as a poor prognostic factor for MPM patients (Supplementary Figures 3, 4C, 5C). Mitotic arrest as a result of KIF11 inhibition has been observed in a variety of tumors (Infante et al., 2012). Several compounds inhibiting KIF11 entered Phase I and II clinical trials (El-Nassan, 2013). Different from KIF11, KIF18B is involved in the regulation of microtubule dynamics (Lee et al., 2010). KIF18B was rarely reported with MPM, but it was reported promoting tumor progression in cervical cancer, hepatocellular carcinoma, and pancreatic cancer (Wu et al., 2018; Yang et al., 2020; Li et al., 2020). High levels of KIF18B were associated with poor prognosis in lung adenocarcinoma patients (Ji et al., 2019). Our analysis showed the high expression of KIF18B was significantly associated with poor prognosis of MPM patients (Supplementary Figures 4D, 5D).

Lysyl oxidase (*LOX*) was one of the five paralogs functioning primarily as the crosslink of collagens or elastin in extracellular matrix (Kim et al., 2011). Studies have shown that LOX mRNA level was increased in various cancer types, including head and neck squamous cell carcinoma, and breast and prostate cancers (Kirschmann et al., 2002; Lapointe et al., 2004; Erler et al., 2006). Recently, LOX was identified as a potential diagnostic biomarker in MPM (Kim et al., 2020). We also note that patients with high expression of LOX had shorter overall survival time and worse prognosis compared with patients with low expression of LOX (Supplementary Figures 4E, 5E).

Several limitations of our study should be pointed out. First, the clinical information of the patients was not comprehensive. Specifically, the clinical information of the discovery set GSE51024 was not accessible. In addition, there was no age, asbestos exposure history or any treatment information for the patients in either GSE2549 or TCGA dataset. Therefore, we could not evaluate additional possible prognostic factors. Second, given the low incidence of MPM, and the scarcity of available public databases, we could only adopt two datasets, GSE2549 and GSE51024, as our discovery datasets. GSE2549 dataset had a split of 40 tumor samples and 9 normal samples, while GSE51024 had 41 pairs of tumor and normal samples, this may lead to result bias and potentially cause the loss of viable DEGs. Third, only one MPM TCGA dataset was used as the validation dataset. The model was constructed based on the DEGs overlapping between the two discovery datasets using microarray technology, yet it was validated in the TCGA dataset using RNA-seq technology which might affect the performance of the model. Compared with RNA-seq, microarray is unable to detect novel transcripts and might cause loss of possible DEGs, particularly genes with low expression. To test the practicality and accuracy of our model, rigorous validation in large prospective studies are needed. Finally, the molecular and biological mechanisms of these five genes in MPM need to be further investigated by additional research.

## Conclusion

In conclusion, we have identified a five-gene signature risk score model as an objective and practical prognostic tool independent of clinicopathological factors for MPM patients, which complements the current MPM staging system. The accuracy and stability of our model provides opportunity for future clinical application.

## Data Availability Statement

Publicly available datasets were analyzed in this study. This data can be found here: https://www.ncbi.nlm.nih.gov/geo/query/acc.cgi?acc=GSE2549, https://www.ncbi.nlm.nih.gov/geo/query/acc.cgi?acc=GSE51024, and https://www.cancer.gov/about-nci/organization/ccg/research/structural-genomics/tcga.

## Author Contributions

YB, XW, JH, LG, KN, and LJ contributed to the study design. YB, XW, XL, ZR, LJ, and KN contributed to the statistical analysis. YB, ZR, and HG contributed to the manuscript draft. YB, XW, LJ, and KN prepared the figures and tables. All authors reviewed and approved the final manuscript and carried out in collaboration work.

## Conflict of Interest

The authors declare that the research was conducted in the absence of any commercial or financial relationships that could be construed as a potential conflict of interest.

## Footnotes

1 https://www.ncbi.nlm.nih.gov/geo

2 https://www.cancer.gov/about-nci/organization/ccg/research/structural-genomics/tcga

3 https://www.gsea-msigdb.org/gsea/index.jsp

## Abbreviations

AJCC: American Joint Commission on Cancer
AUC: area under curve
CI: confidence interval
DEGs: differentially expressed genes
GEO: Gene Expression Omnibus
GSEA: Gene Set Enrichment Analysis
HR: hazard ratio
IMIG: International Mesothelioma Interest Group
MPM: malignant pleural mesothelioma
OS: overall survival
ROC: receiver operating characteristic curve
SMRP: soluble mesothelin-related protein
TCGA: the Cancer Genome Atlas
UICC: Union for International Cancer Control.
