# Supplementary material for: Identification of a Five-Gene Signature for Predicting Survival in Malignant Pleural Mesothelioma Patients
Source: Front Genet. 2020 Aug 7;11:899. doi: 10.3389/fgene.2020.00899 (PMC7427512; doi:10.3389/fgene.2020.00899)
Supplement: Supplementary file 1 [file Data_Sheet_1.docx]

Supplementary Material

**Supplementary Table 1.** Model selection using forward stepwise regression

| Model | AIC |
| --- | --- |
| KIF18B | 175.9 |
| KIF18B+PBK | 174.62 |
| KIF18B+PBK+CKS2 | 172.3 |
| KIF18B+PBK+CKS2+LOX | 170.72 |
| KIF18B+PBK+CKS2+LOX+CDH2 | 170.43 |

Abbreviation: AIC: Akaike information criterion.

**Supplementary Table 2.** AIC and C-index values of different risk score models in GSE2549

|  | Forward stepwise  selection | Stepwise selection | Relaxed LASSO | LASSO |
| --- | --- | --- | --- | --- |
| Genes | KIF18B, PBK, CKS2, LOX, CDH2 | KIF11, KIF18B | CDC20, CKS2, FGF9, FKBP11, KIF11, KIF18B, LOX | CDH2, CKS2, KIF18B, KIF11, LOX |
| AIC | 170.43 | 170.09 | 168.95 | 164.52 |
| C-index | 0.778 (95% CI=0.68588-0.87012) | 0.768 (95% CI=0.65628-0.87972) | 0.786 (95% CI=0.6782-0.8938) | 0.784 (95% CI=0.67424-0.89376) |

Abbreviation: AIC: Akaike information criterion; C-index: the concordance index.

**Supplementary Table 3** AIC and C-index values of different risk score models in TCGA dataset

|  | Forward stepwise  selection | Stepwise selection | Relaxed LASSO | LASSO |
| --- | --- | --- | --- | --- |
| Genes | KIF18B, PBK, CKS2, LOX, CDH2 | KIF11, KIF18B | CDC20, CKS2, FGF9, FKBP11, KIF11, KIF18B, LOX | CDH2, CKS2, KIF18B, KIF11, LOX |
| AIC | 452.74 | 467.07 | 464.17 | 453.95 |
| C-index | 0.747 (95% CI=0.69604-0.79796) | 0.706 (95% CI=0.64132-0.712468) | 0.75 (95% CI=0.68728-0.81272) | 0.753 (95% CI=0.69812-0.80788) |

Abbreviation: AIC: Akaike information criterion; C-index: the concordance index.


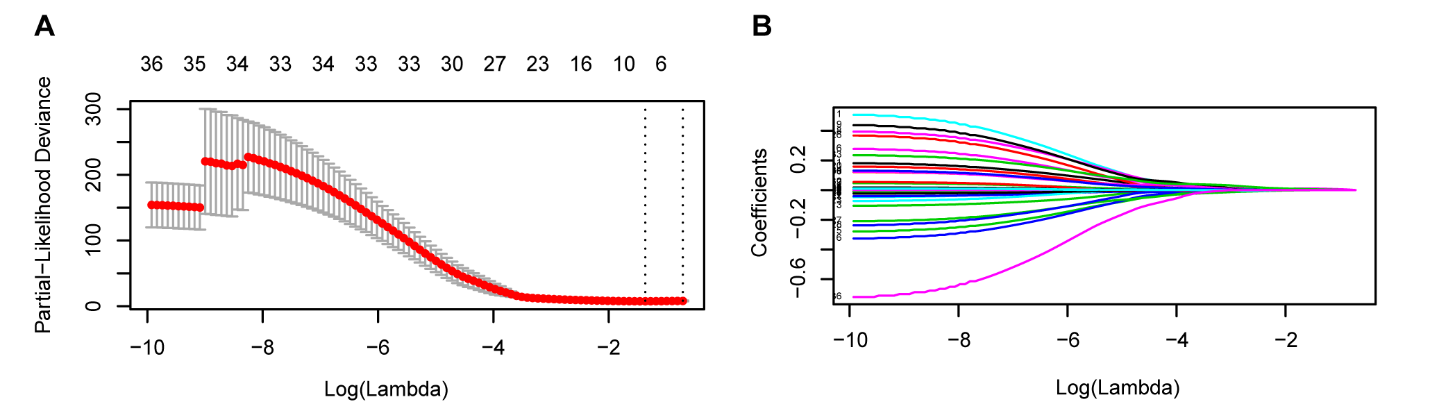
**Supplementary Figure 1. Construction of the risk score model by relaxed lasso method. (A)** Plots of the cross-validation error rates. The two vertical dotted line represent the largest lambda value with minimum error (left) and 1- standard error (right); **(B)** Relaxed LASSO coefficient profiles of the 36 genes detected by univariate cox regression analysis.


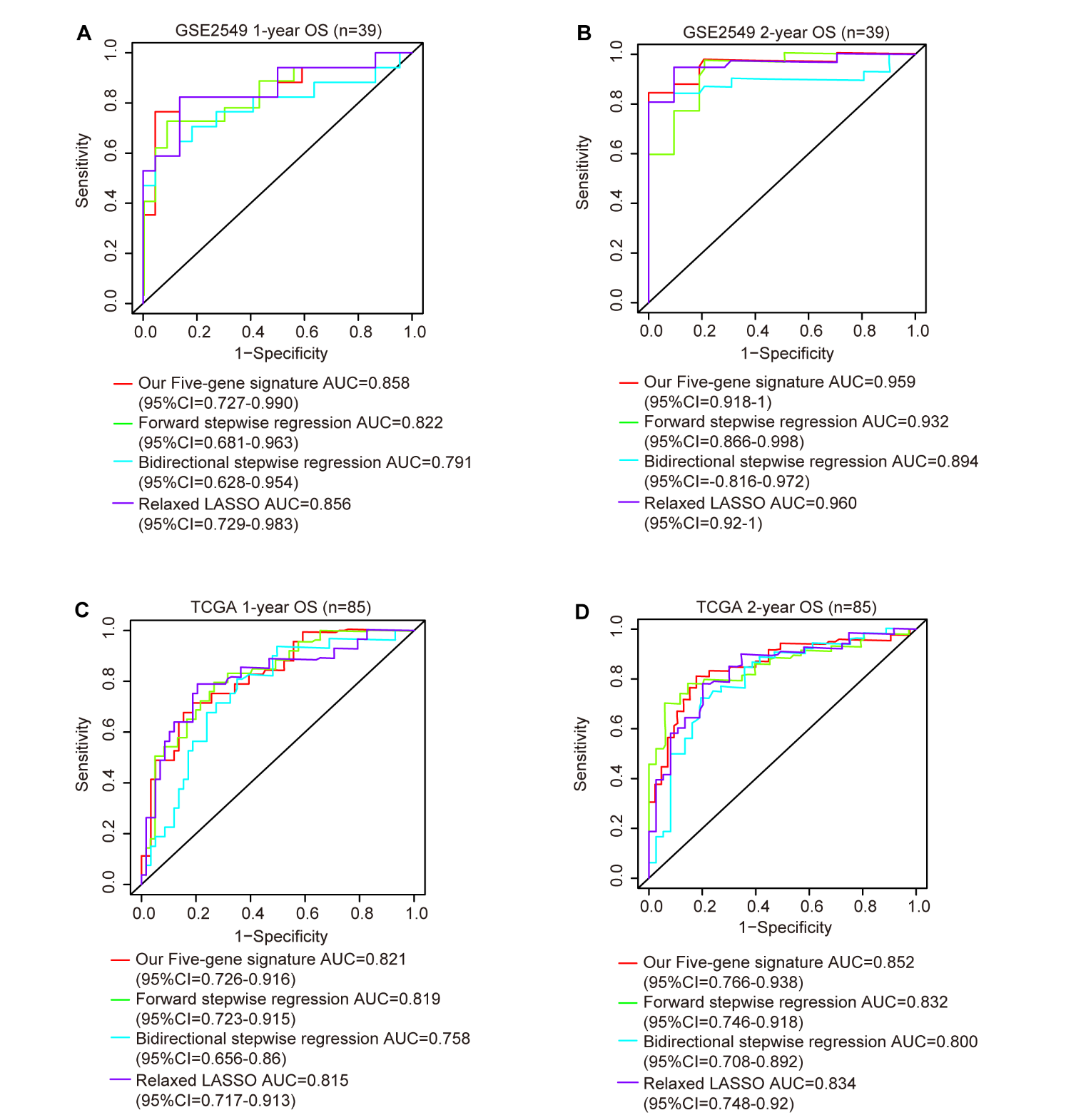


**Supplementary Figure 2. ROC curves our five-gene signature compared with other models constructed by different methods.** The X axis indicates false positive rate. The Y axis indicates true positive rate. One patient in GSE2549 was not included in the analysis because the survival time and vital status were not available. **(A)** 1-year OS in GSE2549; **(B)** 2-year OS in GSE2549; **(C)** 1-year OS in TCGA; **(D)** 2-year OS in TCGA.


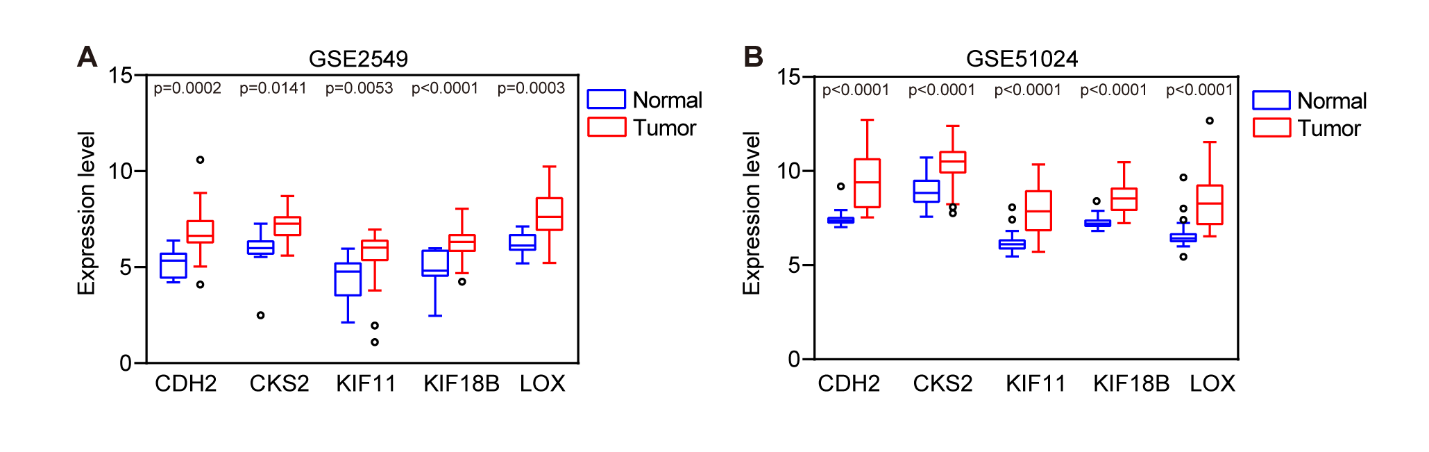


**Supplementary Figure 3. The expressions of the selected five genes in MPM tumor tissues versus normal tissues.** The expression level of the five genes is significantly higher in tumor tissues than normal tissues analyzed by t-test. (**A)** In GSE2549 dataset, the p-value for CDH2, CKS2, KIF11, KIF18B and LOX were 0.0002, 0.0141, 0.0053, <0.0001 and 0.0003, respectively; **(B)** In GSE51024 dataset, the p-value for CDH2, CKS2, KIF11, KIF18B and LOX were all less than 0.0001.

.


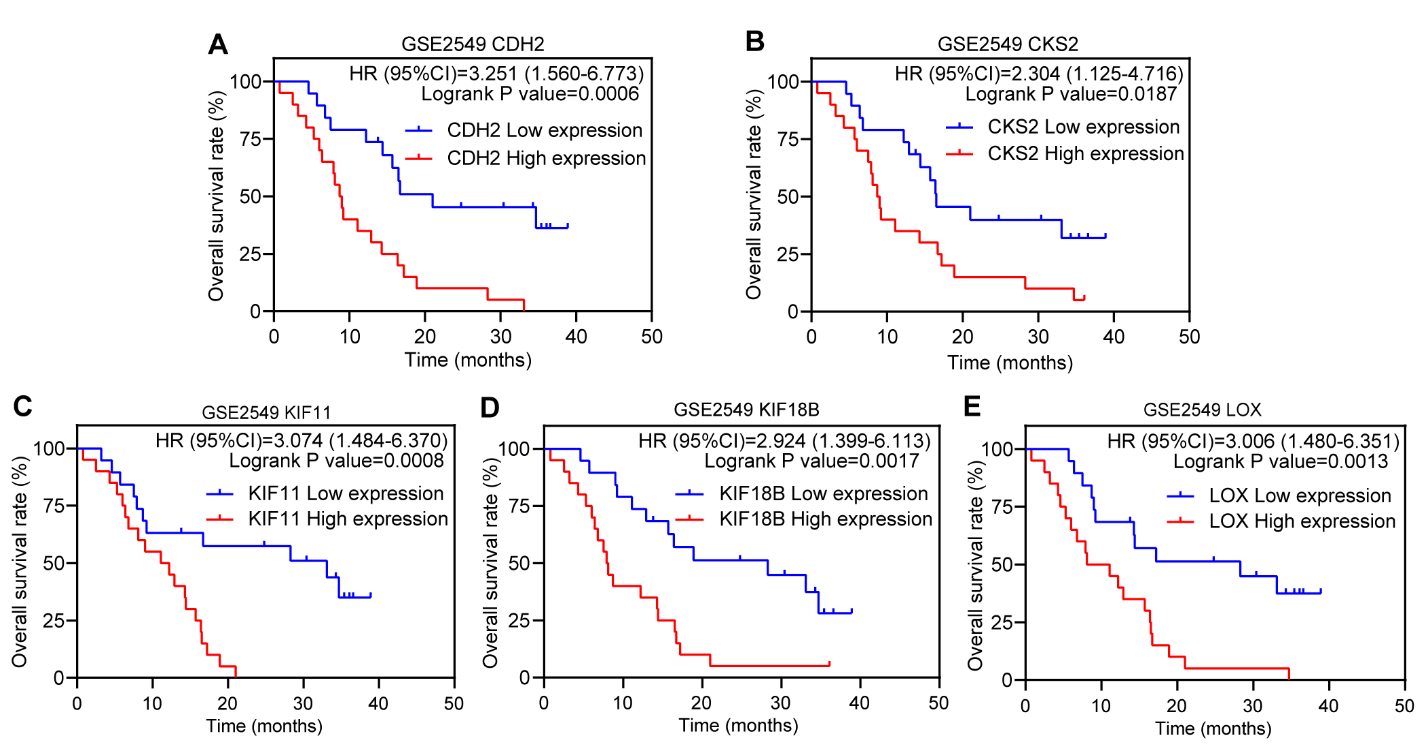
**Supplementary Figure 4.** **Five genes were associated with patients’ OS in GSE2549 dataset by Kaplan-Meier analysis using Log-rank test.** (**A**) CDH2; (**B**) CKS2; (**C**) KIF11; (**D**) KIF18B; (**E**) LOX.


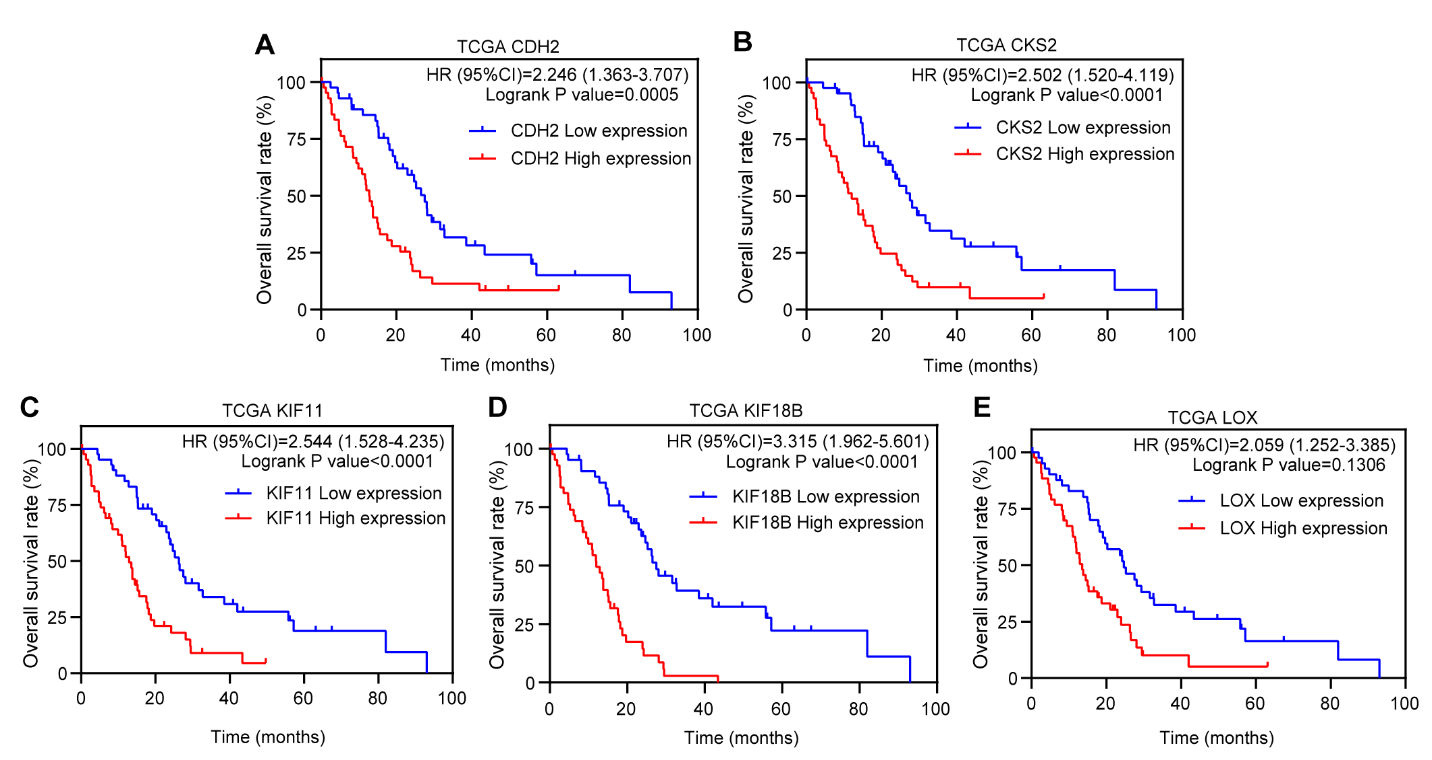
**Supplementary Figure 5. Five genes were associated with patients’ OS in TCGA dataset by Kaplan-Meier analysis using Log-rank test. (A)** CDH2; **(B)** CKS2; **(C)** KIF11; **(D)** KIF18B; **(E)** LOX.


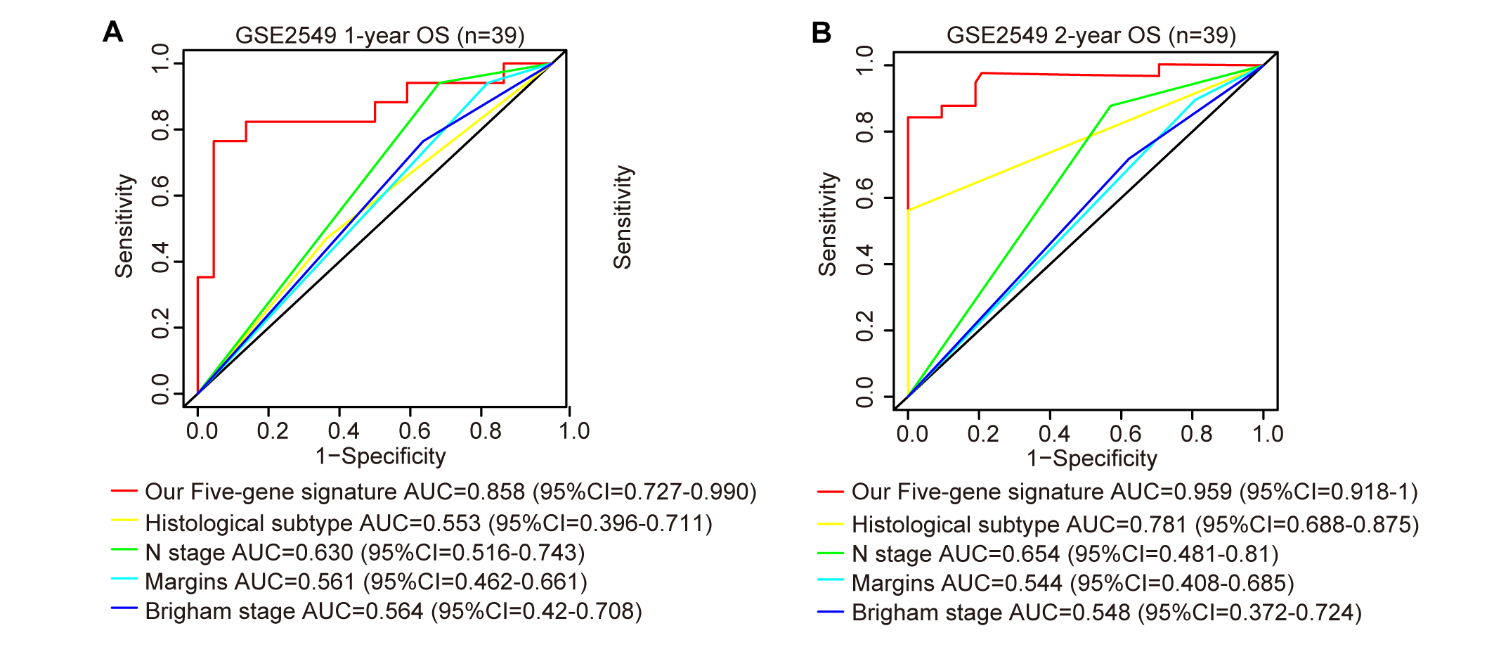


**Supplementary Figure 6. ROC curves of five-gene risk score model compared with clinicopathological factors in GSE2549 dataset.** The X axis indicates false positive rate. The Y axis indicates true positive rate. One patient in GSE2549 dataset was not enrolled in the analysis because the clinical information was not available. **(A)** Comparison with four clinicopathological factors of 1-year OS; **(B)** Comparison with four clinicopathological factors of 2-year OS.


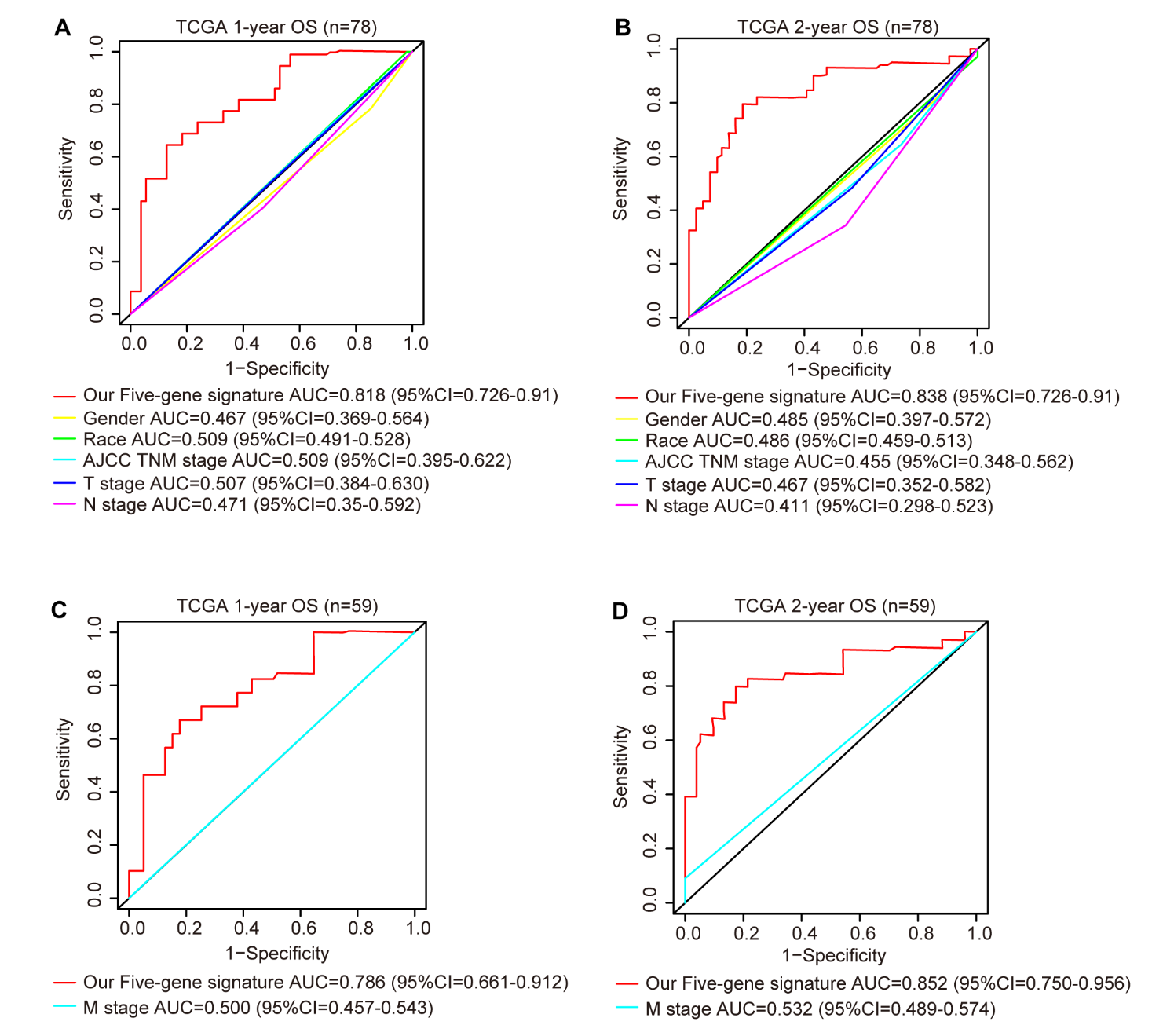


**Supplementary Figure 7. ROC curves of five-gene risk score model compared with clinicopathological factors in TCGA dataset.** The X axis indicates false positive rate. The Y axis indicates true positive rate. Seven patients without complete clinical information were not enrolled in analyses of “gender”, “race”, “AJCC TNM stage”, “T stage” and “N stage”. Twenty-six patients without “M stage” information were not enrolled in “M stage” analysis. **(A)** Comparison with five clinicopathological factors of 1-year OS; **(B)** Comparison with five clinicopathological factors of 2-year OS; **(C)** Comparison with M stage of 1-year OS; **(D)** Comparison with M stage of 2-year OS.


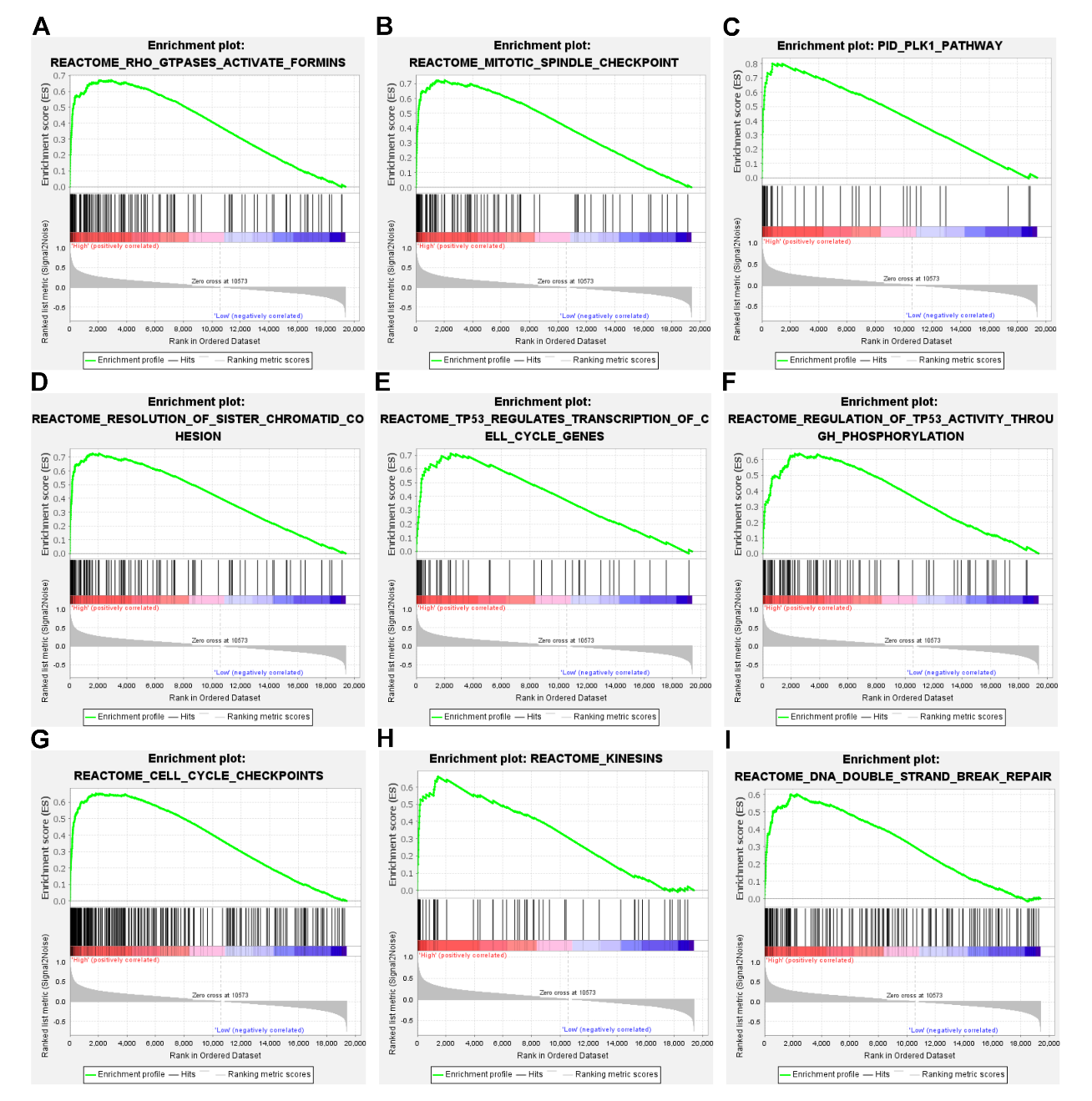


**Supplementary Figure 8. The GSEA analysis results of pathways enriched in high-risk group patients classified by the five-gene signature risk score model.** |NES| (normalized enrichment score) > 1, NOM P-value<0.05 and FDR (false discovery rate) q-value<0.05 after performing 1000 permutations. **(A)** “RHO GTPASES activate formins”; **(B)** “Mitotic spindle checkpoint”; **(C)** “PLK1 pathway”; **(D)** “resolution of sister chromatid cohesion”; **(E)** “TP53 regulates transcription of cell cycle genes”; **(F)** “regulation of TP53 activity through phosphorylation”; **(G)** “cell cycle checkpoints”; **(H)**“Kinesins”; **(I)** “DAN double strand break repair”.
